# *GNAI3*: Another Candidate Gene to Screen in Persons with Ocular Albinism

**DOI:** 10.1371/journal.pone.0162273

**Published:** 2016-09-08

**Authors:** Alejandra Young, Uma Dandekar, Calvin Pan, Avery Sader, Jie J. Zheng, Richard A. Lewis, Debora B. Farber

**Affiliations:** 1 Stein Eye Institute and Department of Ophthalmology, David Geffen School of Medicine, UCLA, Los Angeles, CA, United States of America; 2 Molecular Biology Institute, UCLA, Los Angeles, CA, United States of America; 3 Brain Research Institute, UCLA, Los Angeles, CA, United States of America; 4 UCLA-GenoSeq Core, UCLA, Los Angeles, CA, United States of America; 5 Department of Molecular and Human Genetics, Baylor College of Medicine, Houston, TX, United States of America; University of Florida, UNITED STATES

## Abstract

Ocular albinism type 1 (OA), caused by mutations in the *OA1* gene, encodes a G-protein coupled receptor, OA1, localized in melanosomal membranes of the retinal pigment epithelium (RPE). This disorder is characterized by both RPE macro-melanosomes and abnormal decussation of ganglion cell axons at the brain’s optic chiasm. We demonstrated previously that Oa1 specifically activates Gαi3, which also signals in the Oa1 transduction pathway that regulates melanosomal biogenesis. In this study, we screened the human *Gαi3* gene, *GNAI3*, in DNA samples from 26 patients who had all clinical characteristics of OA but in whom a specific mutation in the *OA1* gene had not been found, and in 6 normal control individuals. Using the Agilent HaloPlex Target Enrichment System and next-generation sequencing (NGS) on the Illumina MiSeq platform, we identified 518 variants after rigorous filtering. Many of these variants were corroborated by Sanger sequencing. Overall, 98.8% coverage of the *GNAI3* gene was obtained by the HaloPlex amplicons. Of all variants, 6 non-synonymous and 3 synonymous were in exons, 41 in a non-coding exon embedded in the 3’ untranslated region (UTR), 6 in the 5’ UTR, and 462 in introns. These variants included novel SNVs, insertions, deletions, and a frameshift mutation. All were found in at least one patient but none in control samples. Using computational methods, we modeled the GNAI3 protein and its non-synonymous exonic mutations and determined that several of these may be the cause of disease in the patients studied. Thus, we have identified *GNAI3* as a second gene possibly responsible for X-linked ocular albinism.

## Introduction

X-linked ocular albinism type 1, historically called the Nettleship-Falls type, has been viewed as the most common form of ocular albinism. It has an estimated prevalence of 1 in every 50,000 live births in the USA. This disorder occurs almost exclusively in males and is characterized by early onset nystagmus, iris transillumination, blond or relatively hypopigmented fundus color, congenital hypoplasia of the fovea centralis, and reduced visual acuity. On careful comparison, most of these affected males have subtlety lighter hair and skin than their unaffected siblings of either gender. Female carriers have a distinctively “mottled” fundus which has no effect on their visual acuity. OA is also characterized by the presence of markedly enlarged melanosomes in the retinal pigment epithelium (RPE) and by abnormal crossing of optic axons at the optic chiasm of affected individuals. The *OA1* gene has been isolated from human [1] and mouse [2], and its transcript encodes a G-protein-coupled receptor (GPCR) [3, 4] localized in RPE melanosomal membranes. Deletion of *Oa1* [5] from the mouse genome results in knockout animals that present similar abnormal phenotypes to those observed in humans with ocular albinism.

In previous work, we showed conclusively by *in-vitro* and *in-vivo* studies in mice that the OA1 protein specifically interacts and activates only one of three heterotrimeric Gαi proteins, Gαi3 (guanine nucleotide binding protein, alpha inhibiting activity polypeptide 3) [6, 7]. This protein signals in the same transduction pathway controlled by OA1 and regulates directly or indirectly the biogenesis of melanosomes (both size and density) in the RPE, as well as axonal guidance through the optic chiasm. Further, we demonstrated that neither Gαi1 nor Gαi2 is involved in these processes. Deletion of *Gαi3* from the mouse genome results in knockout mice with an abnormal RPE phenotype similar to that of *Oa1*-/- mice. Both *Oa1*-/- and *Gαi3*-/- mice have large melanosomes in their RPEs, ~3.8 and 2.3 times larger, respectively, than the largest melanosomes in the corresponding wild type animals, and reduced melanosomal density (number of melanosomes/RPE μm^2^) than control mice [7]. In addition, the size of the uncrossed pathway at the brain’s optic chiasm in both *Oa1*-/- and *Gαi3*-/- mice, obtained by counting the number of ipsilaterally projecting retinal ganglion cells (RGCs), is reduced by 21% in *Oa1*-/- and 12% in *Gαi3*-/- from those of each control mice [6]. Thus, there may be a correlation between abnormal pigmentation in the eye and axon crossing at the optic chiasm, since defective Oa1 and Gai3 protein signaling in embryonic development seems to produce alterations within the RPE cells that are transmitted, possibly through gap junctions, to RGCs as they originate at the ventricular surface of the retina.

It is well established that, in addition to their important roles in many pathways of transmembrane signaling, heterotrimeric G-proteins are localized to the Golgi complex [8] where they are involved in the formation of secretory vesicles that are later released from the trans-Golgi network (TGN) [9]. Gαi3, in particular, acts as an inhibitor of intra-Golgi and post-Golgi trafficking [10]. Its specific function in the RPE is unknown, but based on our prior studies, we hypothesize that Gαi3 controls the size of melanosomes through the inhibition of vesicle trafficking from the TGN to the melanosome [7], a function previously assigned to OA1 [11]. If this were the case, mutations in the *Gαi3* gene of humans (*GNAI3*) could render the OA1 protein unable to activate the non-functional GNAI3 protein on the surface membrane of the melanosome. Without GNAI3 inhibition of the vesicular traffic of melanin-related proteins to the melanosomes, the continuous supply of this material would result in the formation of large organelles. This could explain the presence of macromelanosomes in the RPE of persons with ocular albinism in whom a pathogenic mutation in *OA1* has not been found [12–14]. To test our hypothesis, we have sought mutations in *GNAI3* in DNA samples from 26 patients diagnosed with ocular albinism according to clinical parameters but in whom no *OA1* mutations had been detected by standard CLIA laboratory sequence analyses.

## Materials and Methods

### Human genomic DNA samples

DNA samples from 5 healthy subjects without a personal or family history of ocular albinism (control DNAs) and from 26 anonymized patients diagnosed with ocular albinism but no pathogenic mutation in *OA1* were analyzed for this study. In addition, a sixth control human DNA sample was provided by Agilent as reference. The patient DNA samples had been tested previously for mutations in the *OA1* gene at The Baylor Medical Genetics Laboratory, Baylor College of Medicine, Houston, Texas. Transfer of the patient DNA samples from the Medical Genetics Laboratory at Baylor College of Medicine to DBF’s laboratory at Jules Stein Eye Institute, UCLA was approved by the Institutional Review Board for Human Subject Research for Baylor College of Medicine and Affiliated Hospitals, Protocol # H-28532.

### Capture HaloPlex target enrichment system

A library of DNA restriction fragments from all coding exons, introns and UTRs (5’ and 3’) of the *GNAI3* gene was prepared with a HaloPlex target enrichment kit (Agilent Technologies, Santa Clara, CA, USA), following the manufacturer's instructions. Briefly, 225 ng of genomic DNA from each sample diluted with nuclease-free water to a final concentration of 5 ng/μl were digested in eight different reactions, each containing 2 restriction enzymes. The Enrichment Control DNA (ECD) provided by the kit, which contains genomic DNA mixed with an 800-bp PCR product with restriction sites for all the 16 enzymes in the digestion protocol, was treated in the same manner as the genomic DNA and validated the digestion reaction by gel electrophoresis. Successful digestion was indicated by the appearance of three predominant bands at 125, 225, and 450 bp, corresponding to the 800-bp PCR product-derived restriction fragments. Next, a library of HaloPlex probes (oligonucleotides designed to hybridize selectively to both ends of the genomic DNA restricted fragments and to direct their circularization) was hybridized to the library of genomic DNA restriction fragments. All eight digestion reactions corresponding to each DNA sample were transferred into the appropriate hybridization reaction tube. During the hybridization process, Illumina sequencing motifs including index sequences were incorporated into the targeted fragments. Since the HaloPlex probes were biotinylated, after the hybridization magnetic streptavidin beads retrieved the targeted fragments. DNA ligase was added to the bead-bound samples to close nicks in the circularized HaloPlex probe-target DNA hybrids and 50 mM NaOH was used to elute the captured DNA libraries. Finally, 20 μl of supernatant from each tube containing the enriched DNA fragments were PCR amplified. The target libraries were purified immediately, and their enrichment was validated by gel electrophoresis followed by next-generation sequencing.

### Next-Generation sequence analysis

Sequencing analysis was carried out at the UCLA Sequencing Core Facility with the standard Illumina MiSeq platform protocol (Illumina, San Diego, CA). Before alignment, 5 bp were trimmed from the start of each read to avoid mismapping resulting from restriction site sequence retention. The 250 bp paired-end reads were aligned against the UCSC hg38 Human Reference Genome (https://genome.ucsc.edu/cgi-bin/hgBlat) by the Burrows-Wheeler Aligner [15], BWA version 0.7.12-r1039. Variant calling was performed with LoFreq version 2.1.2 (http://csb5.github.io/lofreq/) with default parameters. All the NGS data has been submitted to the NCBI Sequence Read Archive (SRA) and has accession # SRP074333.

### Sanger sequencing and validation of Next-Generation sequence results

Twenty-two sets of primers (forward and reverse) were designed for sequencing the 5’ flanking region, each of the 9 exons, and the 3’ UTR of the *GNAI3* gene in the genomic DNA samples from the 26 patients with reported ocular albinism who did not have a mutation in the *OA1* gene (Table 1) and from the 6 control individuals. While only one set of primers was used for amplification of the 5’ UTR and exons 1 through 8, 14 sets of primers were required to amplify the long, non-coding exon 9/3’UTR. We tested and optimized each primer by PCR using DNA from a normal individual, and the sizes of the PCR products were verified by electrophoresis on 2% agarose gels. Two different PCR programs (#1 or #2), varying only in the temperature of step 3, allowed optimal amplification of the corresponding fragments:

**Table 1:** Primers used in Sanger sequencing for validation of identified HaloPlex/NGS variants in the *GNAI3* gene.

ID Target Region	Sequence	Tm °C	bp Amplified
5' UTR + Exon 1-F	5' CGCTTTCGGTCTCAACTC 3'	53.4	401
5' UTR + Exon 1-R	5' GCCTTCCAAGCGCCTAG 3'	56.3	
Exon 2 F	5' GATAGACATGAAAGCATCACC 3'	53	323
Exon 2 R	5' CCTCTGAATAGCCATCCTC 3'	51.9	
Exon 3 F	5' CATGGTATTGACTTGTGG 3'	47.8	403
Exon 3 R	5' CTACCGTGCACCCACAGAATC 3'	48	
Exon 4 F	5' GTCTCTGTAACAACACCTC 3'	50	497
Exon 4 R	5' GTGCCAAGTCTCCCATTTAC 3'	53	
Exon 5 F	5' TTTGCCACTTAATAACTAGTAA 3'	56.4	469
Exon 5 R	5' TGGAATGTAGTTAGACTGGGA 3'	54.9	
Exon 6 F	5' GGAAGGTGTATGTGTGAC 3'	50	480
Exon 6 R	5' GCAGCACTAAATGGCATTCAC 3'	57.2	
Exon 7 F	5' GAGGACTGACACTCAAC 3'	49.3	436
Exon 7 R	5' TGCCAATGCCACTACCACTG 3'	57.9	
Exon 8 F	5' GTTGAGTTCAGGCAGCTG 3'	53.8	427
Exon 8 R	5' CGTTCATGCTTGTAGCTGC 3'	54.4	
Exon 9/3' UTR F1	5' GTGCCACAGACACGAAGAATG 3'	56.4	420
Exon 9/3' UTR R1	5' GTGATGTCTTGACGATCG 3'	50.4	
Exon 9/3' UTR F2	5' GGAATGGCAGCAGCATGCAG 3'	59.8	383
Exon 9/3' UTR R2	5' GATCTGGTCACATCATGTGC 3'	53.6	
Exon 9/3' UTR F3	5' CCTTCTTAAACCACCAGTG 3'	50.6	409
Exon 9/3' UTR R3	5' GGTAATCTGCACAAACAAGG 3'	51.7	
Exon 9/3' UTR F4	5' CCTGCTCAAAGTACCATTATG 3'	51.1	425
Exon 9/3' UTR R4	5' AAACGATGGCAAACAGG 3'	50.6	
Exon 9/3' UTR F5	5' CCAGTGACTTTGCTGCTAC 3'	53.6	325
Exon 9/3' UTR R5	5' CTGCCGCCAACTATAAC 3'	50.5	
Exon 9/3' UTR F6	5' CTGTAGTAATCCTTAGCCAG 3'	49.4	380
Exon 9/3' UTR R6	5' GTGAACGTAACTTTCCACAC 3'	51.5	
Exon 9/3' UTR F7	5' GCCTTTGCAGAATGTGCTTTA 3'	59.3	433
Exon 9/3' UTR R7	5' GCACTGAACTGCTATGACTTG 3'	56.5	
Exon 9/3' UTR F8	5' GGAAGACAAATGAAGAGAATG 3'	52	479
Exon 9/3' UTR R8	5' CCATTTCATTAGTCCACAG 3'	48	
Exon 9/3' UTR F9	5' GGTTGGTAGTTGTTACTC 3'	47.1	336
Exon 9/3' UTR R9	5' GGATGGTGAAATAAGAGTG 3'	47.7	
Exon 9/3' UTR F10	5' TTTTCTGCCTCTGTACC 3'	48.6	329
Exon 9/3' UTR R10	5' CAGCAGGAGAATAGCATG 3'	50.2	
Exon 9/3' UTR F11	5' AGTCTCGCTCTGTCGCC 3'	57.2	462
Exon 9/3' UTR R11	5' CAGCATTCTTCAGAGGA 3'	48.4	
Exon 9/3' UTR F12	5' CAAAGAAGCTGAAAGTTGCC 3'	52.5	272
Exon 9/3' UTR R12	5' CAAACTGTCCAAGATGA 3'	46.2	
Exon 9/3' UTR F13	5' GCAACAGATTTCACCTCC 3'	51	376
Exon 9/3' UTR R13	5' CCAAAGAATGTCACAGC 3'	48.3	
Exon 9/3' UTR F14	5' TGTAAGACTAGATGGAC 3'	43.9	460
Exon 9/3' UTR R14	5' CCACAAGTACTAACGCTC 3'	50	

1. 95°C for 3 min; 2. 95°C for 45 sec; 3. #1, 58°C for 45 sec; #2, 47°C (50°C for set 5) for 45 sec; 4. 72°C for 1 min; 5. Repeat 34 times steps 2–4. 6. 72°C for 5 min. For PCR products with two or more amplified bands, the desired band size was cut from the gel and its DNA was extracted with the GenElute Gel Extraction kit (Sigma Aldrich). The eluted DNA was concentrated and then re-suspended in 12 μl of exotoxin-free water.

### Sanger sequencing analysis

The HaloPlex variants within the *GNAI3* UTRs and exons of the 26 patients and 6 control individuals were validated with the Mutation Surveyor’s alignment algorithm (SoftGenetics, State College, PA) which compares the amplified Sanger sequences with those of the NCBI Reference Sequence (RefSeq) database [16]. Candidate variants were identified and shown in electropherograms.

### Analysis of the *GNAI3* non-coding exon 9/3’ UTR’s Regulatory Elements and miRNAs binding sites

We used RegRNA 2.0, an integrated web server, to identify the 3’UTR regulatory elements and miRNAs binding sites in the *GNAI3* gene. The prediction function of this server was selected and the data were obtained in the following three steps: first, we introduced the *GNAI3* mRNA accession number (NM_006496) and pasted its sequence in the FASTA format; second, we selected “mature RNA sequence”; and third, from the types of RNA motifs, we chose the 3 UTR’ regulatory sequences and the miRNA target sites. RegRNA presented the predicted results in both graph and text formats, providing the name, identifier, position in the mRNA sequence, and detailed information of all motifs [17]. To correlate the location of the identified motifs in the mRNA with the corresponding position in the *GNAI3* genomic DNA sequence, we imported the FASTA format of the *GNAI3* mRNA (NM_006496) into the UCSC (GRCh38/hg38) BLAT browser, and then verified whether these motifs were mutated using the specific positions and sequences of SNVs, deletions, and insertions found in the genetic screening of *GNAI3*.

### Computational model of GNAI3 and its non-synonymous mutations

We established computational models of the D102E, V109F, F223V and H213L non-synonymous variants of *GNAI3* to determine how each one of them affects the function of the protein. For this, a structural model of GNAI3 (Protein Data Bank code 2V4Z) [18] in complex with Gβ and Gγ subunits was constructed using a homologous model, Giα_1_β_1_γ_2_, Protein Data Bank code 1GP2 [19] as a template. Amino-acid sequence alignment and loop optimization were performed with the Prime module of the Schrödinger suite, and point mutations were made with Swiss-PdbViewer. Model structures were visualized with PyMOL.

## Results

### Next-generation sequencing findings

With the capture HaloPlex target enrichment system, we sequenced the entire *GNAI3* gene (~47Kb) on the Illumina MiSeq platform from the DNA samples of the 26 patients and six normal control individuals. We obtained >20 million usable 250-bp reads. Quality trimming and sequence alignment used the Agilent SureCall and BWA softwares [15]. To ensure the identification of all possible significant variants that could lead to disease, we used LoFreq version 2.1.2, a fast and sensitive program for inferring both single nucleotide variants (SNVs) and insertions/deletions (indels). LoFreq makes full use of base-call qualities and other sources of errors inherent in sequencing (e.g., mapping or base/indel alignment uncertainty), which are usually ignored by other methods. Each variant call is assigned a p-value which allows for rigorous false positive control [20]. After calling all variants, those from control DNAs were filtered from those of patients’ DNAs. These were filtered further with a Phred-scaled quality score cutoff of 40 and a minimum sequencing depth of 100. Also, the sum of the observed bases divided by the sequencing depth had to exceed 90%.

During this work, we found that the position and size of the *GNAI3* gene in Ensembl Homo sapiens GRCh37/hg19 coordinates, mapped to Chr1:110,091,186–110,138,465 (genomic size 47,279 bp), was changed to Chr1:109,548,611–109,618,321 (genomic size 69,710 bp) in the updated GRCh38/hg38 Homo sapiens Ensembl. *GNAI3* is composed of 9 exons, 8 of which are coding (1–8) and one is non-coding (exon 9). The size of all coding exons and introns is the same in the two Homo sapiens Ensembl sequences, but exon 9 is embedded in the 3’ UTR, and together they cover 25,977 bp in the GRCh38 version. The position of all exons and introns and their lengths in the updated human genome sequence is shown as Table 2.

**Table 2:** Summary of *GNAI3* gene exons, introns and 5’ and 3’ UTR sequences (ENST00000369851) in the 5' to 3' direction (Ensembl GRCh38/hg38).

No.	Exon / Intron^+^	Start	End	Length	Sequence
	**5' upstream sequence**	** **	** **	** **	**..........gccggaagtgtcgtaaacgtcggatatccggttcttctgggcgctaaggg**
**1**	**ENSE00001451089**	109,548,611	109,548,838	228	AGCTGACGGAGAGGGCCACCGCCCAGCAATAGACGGTGCCTCAGC….
** **	Intron 1–2	109,548,839	109,573,736	24,898	gtgaggggctggaggcggggactga..........aaagtctggttttcttttcttacag
**2**	**ENSE00001781395**	109,573,737	109,573,779	43	GTGCTGGAGAATCTGGTAAAAGCACCATTGTGAAACAGATGAA
** **	Intron 2–3	109,573,780	109,573,895	116	gtaagttggaatgtagcgttttgtt..........acttgtggttttctttgttttaaag
**3**	**ENSE00000783439**	109,573,896	109,574,037	142	AATCATTCATGAGGATGGCTATTCAGAGGATGAATGTAAAC….
** **	Intron 3–4	109,574,038	109,579,203	5,166	gtaagtgtttctcatttcctcttca..........tctctttcttaactgctttcttcag
**4**	**ENSE00000826739**	109,579,204	109,579,361	158	GATGATGCCCGGCAATTATTTGTTTTAGCTGGCAGTGCTGAAGAAGG…
** **	Intron 4–5	109,579,362	109,582,436	3,075	gtaagtaatttttctctgtgaaact..........aaaagtaaacgtgtcttttatttag
**5**	**ENSE00000826740**	109,582,437	109,582,565	129	TTATCTAAATGATCTGGATAGAATATCCCAGTCTAACTACATTCCAACT…
** **	Intron 5–6	109,582,566	109,586,215	3,650	gtaagtcattagcctttttgctagg..........atttgctttctttccccttgcgcag
**6**	**ENSE00001740751**	109,586,216	109,586,345	130	GATGTTTGATGTAGGTGGCCAAAGATCAGAACGAAAAAAGTGGATT…
** **	Intron 6–7	109,586,346	109,586,728	383	gtatgttggagcttctggtaaaaag..........ctatcatcctttatttctttttcag
**7**	**ENSE00001756567**	109,586,729	109,586,882	154	AACCGAATGCATGAAAGCATGAAACTGTTTGACAGCATTT….
** **	Intron 7–8	109,586,883	109,592,042	5,160	gtaaggggttatgaaagattttatt..........tgagagtactgggtgtccgttttag
**8**	**ENSE00000913041**	109,592,043	109,592,255	213	GTTCCAATACATATGAAGAGGCAGCTGCCTATATTCAATGCCA….
** **	Intron 8–9	109,592,256	109,592,344	89	gtaaagtttcatacaaggtagttat..........aattaagaatattcttctcttctag
**9**	**ENSE00001342841**	109,592,345	109,618,321	25,977	TTACTACAGTGTGGAGTGTTGAGACCAGACACCTTTTGCTGTCTCA….
	**3' downstream sequence**	** **	** **	** **	**caatttttaatggctgtatttcatggtatgaatattacataaacattccc..........**

^**+**^Exons are depicted in uppercase letters and introns and UTRs in lowercase letters.

**Fig 1:**
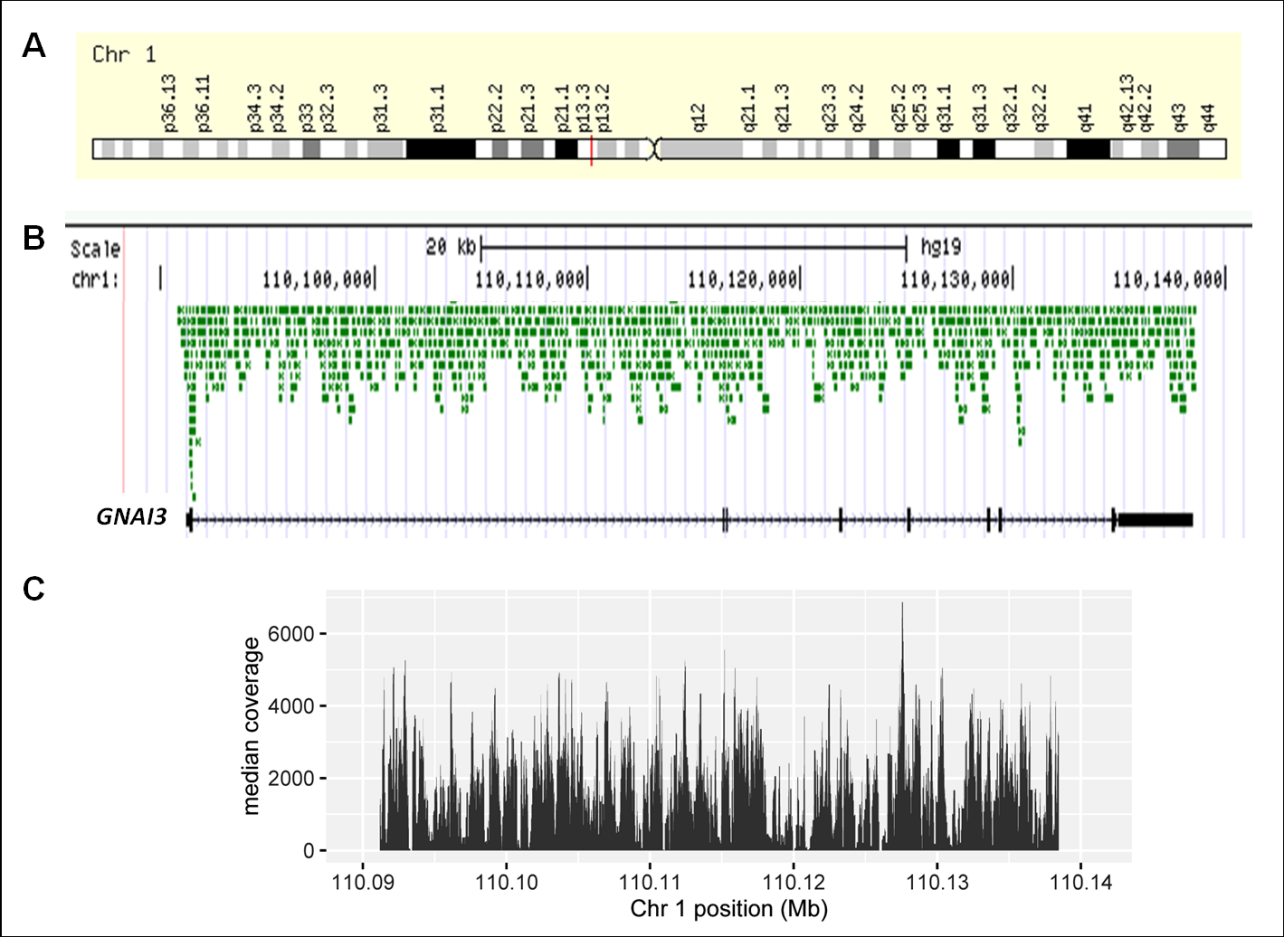
*GNAI3* gene locus in human Chr1, Agilent Haloplex amplicons and Median coverage of *GNAI3*. **A**.*GNAI3* is located in the p arm of chromosome 1, band 13.3 (red line). **B.** Agilent Haloplex Amplicons designed to amplify the *GNAI3* 9 exons, introns and 100 bp of each 5’ and 3’ UTRs are depicted in green and show that all regions of the gene were effectively covered. The *GNAI3* RefSeq is shown in black and was aligned against the GRCh37/hg19 human genome using the UCSC-BLAT browser. **C.** The median coverage level varied from 14 to 8,009 across the targeted *GNAI3* region analyzed in the HaloPlex design; only 1.2% of the target *GNAI3* region was not covered.

Initially, Agilent HaloPlex amplicons were designed from the GRCh37/hg19 human genome sequence. They covered 46,888 bp of the Chr*1* 47,479 bp target region (*GNAI3* genomic size, 47,279, plus 100 bp of each 3’ and 5’ UTRs), resulting in 98.8% coverage of the (earlier version) whole gene. The position of *GNAI3* in Chromosome 1p13.3 is shown as Fig 1A and the base-by base average coverage of the gene by the HaloPlex amplicons, as Fig 1B. The median level of coverage across the entire *GNAI3* gene, 2,023, is shown as Fig 1C and was calculated with the median of the 32 samples (from 26 OA1 patients and 6 controls); the mean average depth of coverage, also calculated with the mean of the 32 samples, was 2,326 +/-1,391.

We found 258 SNVs in the *GNAI3* gene from the subjects with ocular albinism that were also observed in the unaffected, unrelated control DNA samples. After filtering all these common polymorphisms present in control samples, NGS of the *GNAI3* gene identified 6 SNVs in its 5’ UTR (within the promoter and regulatory sequences, Table 3), 1 frameshift deletion in exon 1 and 5 non-synonymous variants in exons 4 and 6 (Table 4), as well as 3 synonymous variants, one in each of exons 1 (c.G105A:p.K35K), 2 (c.T120G:p.G40G) and 3 (c.C222T:p.Y74Y). Many variants (41) were also observed in the non-coding exon 9/3’ UTR, (27 SNVs and 14 indels identified by asterisks in Tables 5 and 6, respectively); but the largest number of SNVs (462) were intronic (data not shown). All resulting alleles identified in this study were present in the DNA of at least one subject with ocular albinism but in none of the six control DNA samples.

**Table 3:** Summary of SNVs in the *GNAI3* 5' UTR of ocular albino patients.

Patient	Chr. 1 Position	Ref	Alt	Variant Detail ^+^	SNP ID
**2**	109548649	C	T	c.-72C>T*	rs3737182
**4**	109548647	T	G	c.-74T>G*	
**9**	109548687	A	G	c.-34A>G*	
**10**	109548660	C	T	c.-61C>T*	rs1279195
** **	109548717	C	T	c.-4C>T*	rs3814308
**13**	109548649	C	T	c.-72C>T	rs3737182
**15**	109548665	C	A	c.-56C>A*	rs144431312
**20**	109548717	C	T	c.-4C>T	rs3814308
**22**	109548649	C	T	c.-72C>T	rs3737182
**25**	109548660	C	T	c.-61C>T	rs1279195
**26**	109548660	C	T	c.-61C>T	rs1279195
** **	109548717	C	T	c.-4C>T	rs3814308

^+^ Variants are listed per patient and not following the 5’ to 3’ order in which they appear in the 5’ UTR.

* identifies the 6 HaloPlex variants in the 5’UTR of *GNAI3*. Some of these variants are present in more than one patient.

**Table 4:** Summary of variants in exons of *GNAI3* with likely functional effects in patients diagnosed with ocular albinism.

Patient	Chr. 1 Position	Ref	Alt	Exon	Type of Variant^+^	AA Change
**2**	109548803	A	-	1	**frameshift deletion**	E28fs
**3**	109579206	T	G	4	**Non-synonymous SNV**	D102E
**7**	109586263	A	T	6	**Non-synonymous SNV**	H213L
**16**	109579226	T	G	4	**Non-synonymous SNV**	V109G
**21**	109579225	G	T	4	**Non-synonymous SNV**	V109F
** **	109586292	T	G	6	**Non-synonymous SNV**	F223V

^+^ Variants are listed per patient and not per exon number.

**Table 5:** Summary of SNVs in the first 2109 bp of the *GNAI3* non-coding exon 9/3’ UTR in patients affected with ocular albinism.

Patient	Chr. 1 Position	Ref	Alt	Variant Detail^+^	SNP ID
**1**	109594018	T	G	uc001dxz.3:c.*1696T>G*	
** **	109594370	T	G	uc001dxz.3:c.*2048T>G*	
**2**	109592363	T	G	uc001dxz.3:c.*41T>G*	
** **	109592686	G	A	uc001dxz.3:c.*364G>A*	rs7371
**3**	109593305	T	G	uc001dxz.3:c.*983T>G*	
** **	109594530	C	T	uc001dxz.3:c.*2208C>T*	
**4**	109592959	G	T	uc001dxz.3:c.*637G>T*	
**6**	109592412	C	T	uc001dxz.3:c.*90C>T*	
** **	109592425	T	G	uc001dxz.3:c.*103T>G*	
** **	109593410	T	G	uc001dxz.3:c.*1088T>G*	
**7**	109594019	T	G	uc001dxz.3:c.*1697T>G*	
** **	109594020	T	G	uc001dxz.3:c.*1698T>G*	
** **	109594370	T	G	uc001dxz.3:c.*2048T>G	
**8**	109592540	G	T	uc001dxz.3:c.*218G>T*	
**9**	109593120	G	A	uc001dxz.3:c.*798G>A*	rs115879755
**10**	109594619	G	T	uc001dxz.3:c.*2297G>T*	
**11**	109592686	G	A	uc001dxz.3:c.*364G>A	rs7371
** **	109593019	T	G	uc001dxz.3:c.*697T>G*	
** **	109594018	T	G	uc001dxz.3:c.*1696T>G	
** **	109594370	T	G	uc001dxz.3:c.*2048T>G	
**12**	109593410	T	G	uc001dxz.3:c.*1088T>G	
** **	109594020	T	G	uc001dxz.3:c.*1698T>G	
** **	109594370	T	G	uc001dxz.3:c.*2048T>G	
**13**	109592686	G	A	uc001dxz.3:c.*364G>A	rs7371
** **	109593019	T	G	uc001dxz.3:c.*697T>G	
** **	109594056	C	T	uc001dxz.3:c.*1734C>T*	rs2301229
** **	109594224	T	A	uc001dxz.3:c.*1902T>A*	rs3525
**14**	109593019	T	G	uc001dxz.3:c.*697T>G	
**15**	109593019	T	G	uc001dxz.3:c.*697T>G	
** **	109594370	T	G	uc001dxz.3:c.*2048T>G	
**16**	109593019	T	G	uc001dxz.3:c.*697T>G	
** **	109593637	T	G	uc001dxz.3:c.*1315T>G*	
**17**	109593410	T	G	uc001dxz.3:c.*1088T>G	
** **	109594262	T	C	uc001dxz.3:c.*1940T>C*	rs41280328
**18**	109593368	T	G	uc001dxz.3:c.*1046T>G*	
** **	109593411	T	G	uc001dxz.3:c.*1089T>G*	
**19**	109593517	T	G	uc001dxz.3:c.*1195T>G*	
**20**	109592506	G	T	uc001dxz.3:c.*184G>T*	
** **	109593243	T	G	uc001dxz.3:c.*921T>G*	
** **	109593305	T	G	uc001dxz.3:c.*983T>G*	
**21**	109594018	T	G	uc001dxz.3:c.*1696T>G	
** **	109594262	T	C	uc001dxz.3:c.*1940T>C	rs41280328
**22**	109592686	G	A	uc001dxz.3:c.*364G>A	rs7371
** **	109593019	T	G	uc001dxz.3:c.*697T>G	
** **	109593500	T	G	uc001dxz.3:c.*1178T>G	
**23**	109593484	G	A	uc001dxz.3:c.*1162G>A*	rs184476515
**24**	109592686	G	A	uc001dxz.3:c.*364G>A	rs7371
** **	109593484	G	A	uc001dxz.3:c.*1162G>A	rs184476515
** **	109594019	T	A	uc001dxz.3:c.*1697T>A	rs41280248
** **	109594224	T	A	uc001dxz.3:c.*1902T>A	rs3525

^+^ Variants are listed per patient and not following the 5’ to 3’ order in which they appear in the exon 9/3’ UTR.

* represents the 27 SNVs in 2109 bp of the non-coding exon 9/3’ UTR identified with HaloPlex/NGS analyses.

**Table 6:** Summary of deletions and insertions in the first 2109 bp of the *GNAI3* non-coding exon 9/3’ UTR in patients affected with ocular albinism.

Patient	Chr. 1 Position	Ref	Alt	GNAI3 Gene	Variant Detail^+^	SNP ID
**2**	109592643	T	-	NC-EX-9	uc001dxz.3:c.*321delT*	
**3**	109594256	G	-	NC-EX-9	uc001dxz.3:c.*1934delG*	
**6**	109593149	A	-	NC-EX-9	uc001dxz.3:c.*827delA*	
**7**	109594646	A	-	NC-EX-9	uc001dxz.3:c.*2324delA*	
**10**	109594316	-	A	NC-EX-9	uc001dxz.3:c.*1994_*1995insA*	
**11**	109593701	AT	-	NC-EX-9	uc001dxz.3:c.*1379_*1380delAT*	rs3833901
** **	109593976	A	-	NC-EX-9	uc001dxz.3:c.*1654delA*	
**12**	109593825	G	-	NC-EX-9	uc001dxz.3:c.*1503delG*	
**13**	109593700	GA	-	NC-EX-9	uc001dxz.3:c.*1378_*1379delGA*	rs3833901
** **	109593701	AT	-	NC-EX-9	uc001dxz.3:c.*1379_*1380delAT	
** **	109593976	A	-	NC-EX-9	uc001dxz.3:c.*1654delA	
**14**	109593462	T	-	NC-EX-9	uc001dxz.3:c.*1140delT*	
**17**	109593493	A	-	NC-EX-9	uc001dxz.3:c.*1171delA*	
** **	109594439	T	-	NC-EX-9	uc001dxz.3:c.*2117delT*	
**18**	109594316	-	A	NC-EX-9	uc001dxz.3:c.*1994_*1995insA	rs144920545
**19**	109592643	T	-	NC-EX-9	uc001dxz.3:c.*321delT	
**22**	109593700	GA	-	NC-EX-9	uc001dxz.3:c.*1378_*1379delGA	
** **	109593701	AT	-	NC-EX-9	uc001dxz.3:c.*1379_*1380delAT	rs3833901
**24**	109593682	T	-	NC-EX-9	uc001dxz.3:c.*1360delT*	
** **	109593701	AT	-	NC-EX-9	uc001dxz.3:c.*1379_*1380delAT	rs3833901
**25**	109592649	C	-	NC-EX-9	uc001dxz.3:c.*328delC*	
**26**	109594316	-	A	NC-EX-9	uc001dxz.3:c.*1994_*1995insA	rs144920545

^+^ Variants are listed per patient and not following the 5’ to 3’ order in which they appear in exon 9/3’ UTR.

* represents the 14 Indels in 2,109 bp of the non-coding exon 9/3’ UTR identified with HaloPlex/NGS analyses.

**Fig 2:**
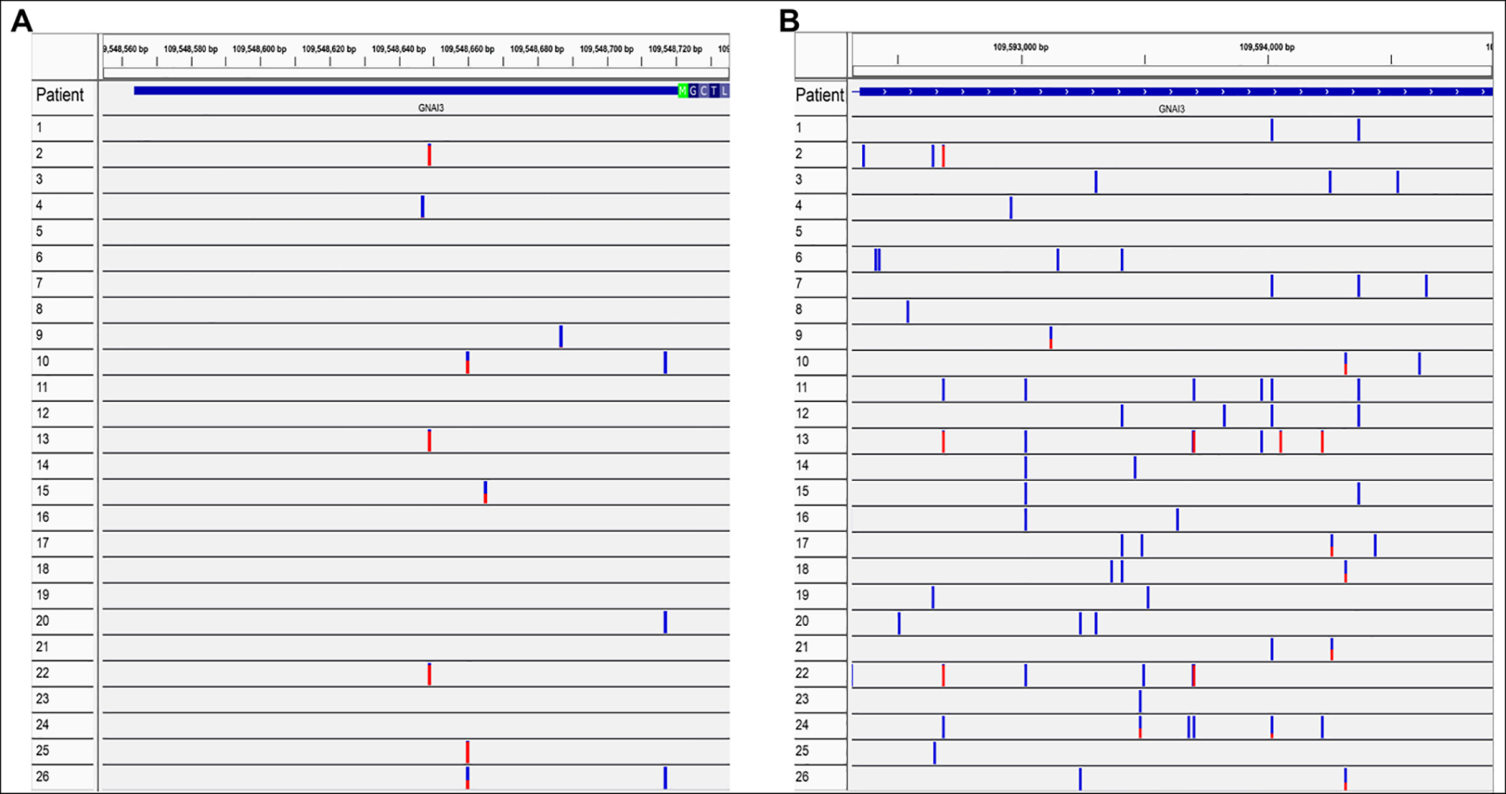
Variants identified in the 5’UTR and non-coding exon 9/3’ UTR using the HaloPlex target enrichment system/IIlumina MiSeq sequencing and visualized with the Integrative Genomics Viewer (IGV). **A.** SNVs found in the 5’ UTR of the *GNAI3* gene, represented by the blue horizontal bar before the start codon encoding Methionine (green), at the beginning of exon 1. **B**. SNVs, insertions and deletions found in the *GNAI3* 2109 bp *of* non-coding exon 9/3’ UTR, represented by the blue horizontal bar. Variants are homozygous [red or blue (lower frequency)] and heterozygous (half blue and half red).

Of the 26 unrelated affected subjects, 10 showed SNVs in the 5’ UTR (Fig 2A). Some of these are homozygous SNVs, such as c.-74T>G in patient 4, c.-72C>T in patients 2, 13, and 22, c.-61C>T in patient 25, c.-34A>G in patient 9, and, c.-4C>T in patients 10, 20, and 26. Heterozygous SNVs included c.-61C>T in patients 10 and 26, and c.-56C>A in patient 15. Non-synonymous SNVs with potential pathological effects are present in 5 of the 26 DNAs from affected subjects that did not have mutations in *OA1* (Table 4). Patient 2 has a frameshift deletion, Patients 3, 7, and 16 have one substantive amino-acid changed (an aspartic acid to glutamic acid, a histidine to leucine, and a valine to glycine, respectively), and patient 21 has two amino-acids changed (a valine to phenylalanine and a phenylalanine to valine).

Analysis of the 9/3’UTR showed that, while some SNVs are exclusively present in one patient, others are shared by up to six patients (Fig 2B). For example, homozygous SNV c.*364G>A (Chr1 position 109592686) is present in patients 2, 11, 13, 22, and 24, whereas heterozygous SNV c*798G>A (Chr1 position 109593120) is seen only in patient 9. Similarly, deletion c.*1379_*1380delAT (Chr1 position 109593703) is present in patients 11, 13, 22, and 24, while deletion c.*328delC (Chr1 position 109592649) is observed only in patient 25. A single insertion is detected in patients 10, 18, and 26, c.*1994_*1995insA (Chr1 position 109594317). All patients show many intronic variants, which are SNVs, deletions, or insertions (Table 7). Patient 3 has the lowest number of them (12). With the exception of Patients 2, 7, 14, and 26, who have 44 to 49 variants each, and Patient 16 that has the highest number (72), each of the other patients has between 18 and 39 intronic variants. The highest number of deletions in introns is nine, in Patient 22, followed by 6 in Patients 2, 4, 15, and 26. The other patients have between one and five deletions. Insertions are not as common. In fact, seven patients do not have any. Patient 25 has the most (eight), followed by Patient 10 with six and Patient 2 with five. All other patients have between one and four insertions. Each patient has his own unique variants and many others are shared with other patients (Table 7).

**Table 7:** Summary of SNVs, deletions and insertions in *GNAI3* introns of patients diagnosed with ocular albinism.

Patient	Total Variants	SNVs	Deletions	Insertions	Unique Variants
**1**	16	13	3	0	4
**2**	44	33	6	5	11
**3**	12	9	3	0	6
**4**	33	27	6	0	10
**5**	19	14	3	2	3
**6**	28	25	3	0	13
**7**	47	43	2	2	17
**8**	17	12	3	2	12
**9**	22	18	3	1	6
**10**	32	22	4	6	7
**11**	39	31	5	3	14
**12**	36	33	1	2	7
**13**	39	34	5	0	12
**14**	49	41	4	4	17
**15**	27	21	6	0	8
**16**	72	65	4	3	34
**17**	21	16	4	1	9
**18**	22	18	3	1	9
**19**	29	23	3	3	8
**20**	18	12	4	2	10
**21**	29	24	4	1	10
**22**	36	25	9	2	12
**23**	16	15	1	0	5
**24**	29	21	5	3	7
**25**	34	23	3	8	16
**26**	48	40	6	2	33

### Sanger Sequencing Validation

**Fig 3:**
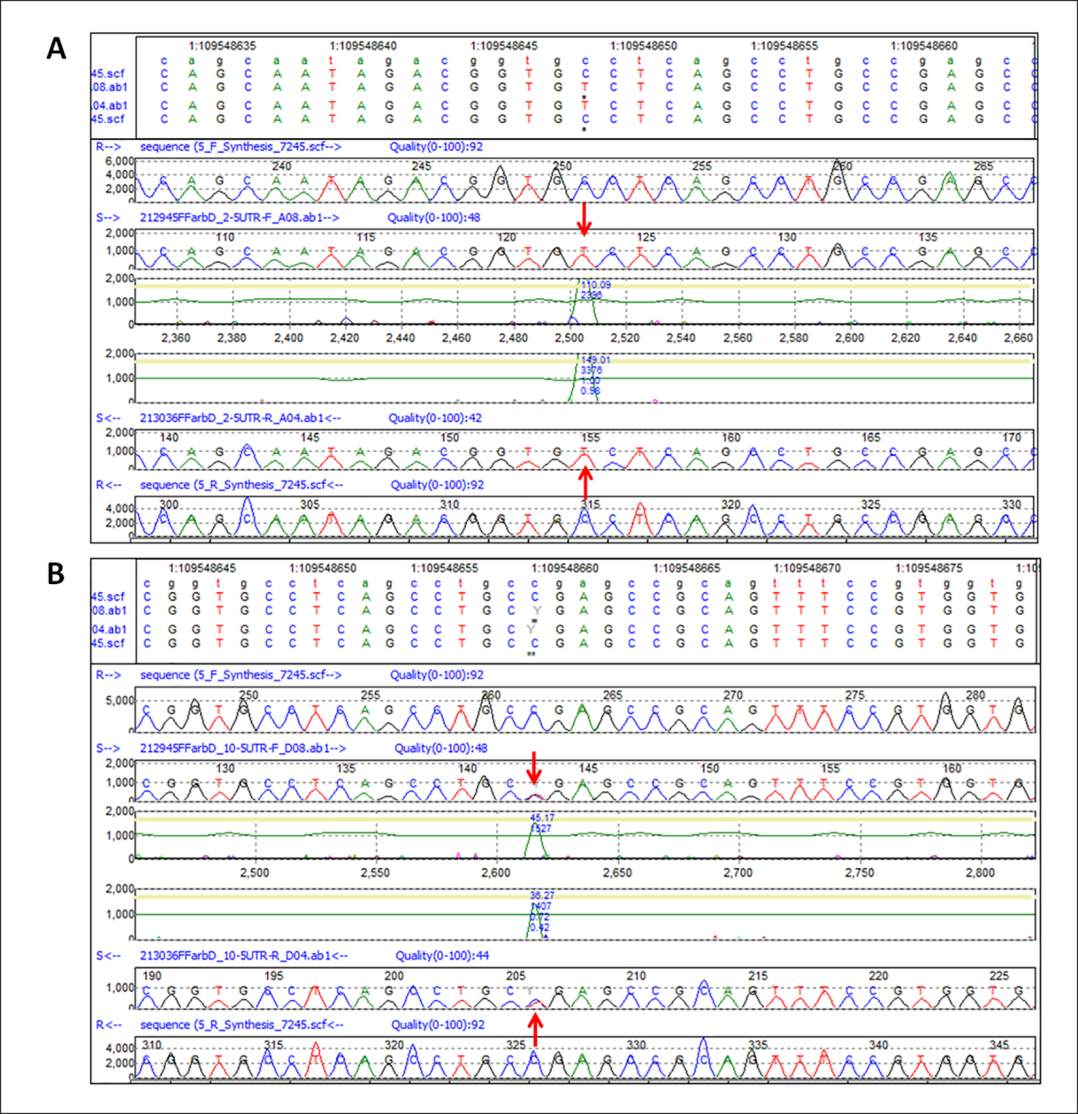
Validation of HaloPlex SNVs in the 5’ UTR of *GNAI3* by Sanger Sequencing. **A.** Homozygous SNV (c.-72C>T) of Patient 2. At the top of the Figure the track annotation panel shows the Chr1 location of the C >T variant at position 109548649. Next, the forward sequences of the reference (R) and test sample (S) are shown, followed by the confidence score of the peak corresponding to the variant. From the bottom up, the reverse sequences of the reference and test sample peak profiles and the confidence score demonstrate the same variant. The red arrows point to the mutated nucleotide. **B.** Heterozygous SNV (c.-61C>T) of Patient 10. Same as **A,** except for the track annotation panel, which shows the C>T SNV at position 109548660 in Chr1.

Variants identified by the HaloPlex Target Enrichment System/NGS were confirmed by Sanger sequencing following PCR amplification of all *GNAI3* exons and their adjacent 10–15 bp, as well as of sequential fragments of the 5’ and 3’ UTRs. The resulting data were compared to the reference sequence of the RefSeq database [16]. Since classical Sanger sequencing allows the detection of mutations only with an allelic frequency of at least 20% [21], quite a few of the significant but low frequency mutations (allele frequency less than 0.03%) identified with LoFreq were not detected with the Sanger method due to its low sensitivity. As examples of the many sequences that we have validated, we show four different electropherograms of sequences containing a homozygous or heterozygous SNV, an insertion or a deletion in the *GNAI3* gene. The electropherogram of a 5’UTR region in Patient 2 (Fig 3A) shows a homozygous SNV (c.-72C>T) at position 109548649 that corroborates the HaloPlex result shown in Fig 2A. Similarly, the electropherogram of a very close 5’ UTR region in Patient 10 (Fig 3B) shows a heterozygous SNV (c.-61C>T) at position 109548660, validating this HaloPlex variant seen in Fig 2A.

**Fig 4:**
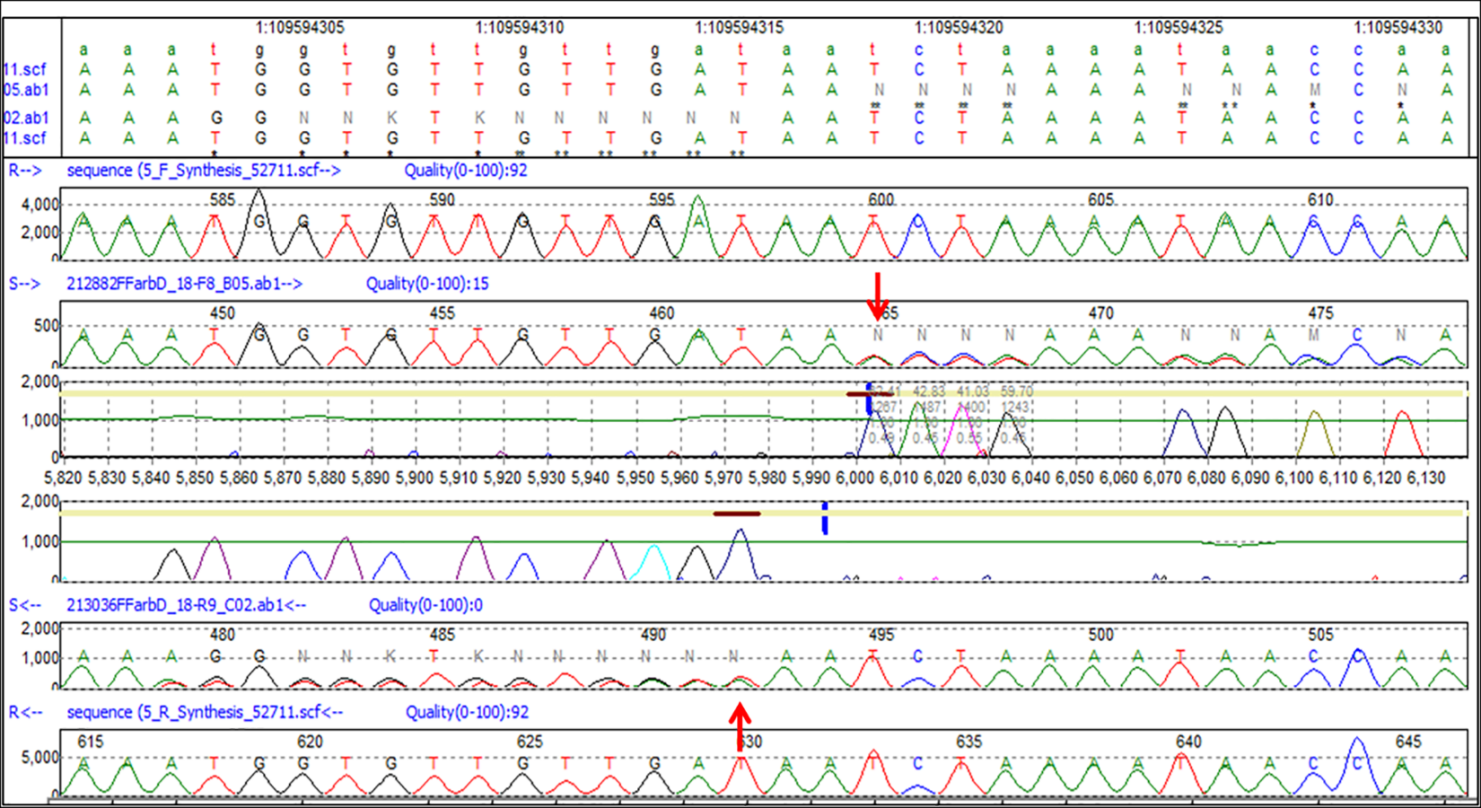
Validation of HaloPlex heterozygous insertion (c.*1994_*1995insA) of Patient 18 in the non-coding exon 9/3’ UTR of *GNAI3* by Sanger Sequencing. As in the previous figures, the track annotation panel depicts the Chr1 location of the insertion at position 109594317, followed by the forward sequences of the reference (R) and test sample (S), the confidence score peaks and reverse reference and test sample sequences. The red arrows point to the insertion in the sequences.

Sanger sequencing also confirmed the HaloPlex heterozygous insertion of an A at position 109594317 in exon 9/3’UTR (Fig 2B) of Patients 10, 18, and 26. The forward and reverse sequences for Patient 18 compared to those of the reference sequence are shown in Fig 4. The red arrows indicate the A insertion, and from this point on the sequence of the mutant allele is shifted by one nucleotide, thereby altering the amino-acid sequence of the translated protein. In addition, analyses of the electropherograms of DNA samples from patients 11, 13, 22, and 24 show a homozygous deletion of AT at position 109593703–109593704 in exon 9/3’UTR.

**Fig 5:**
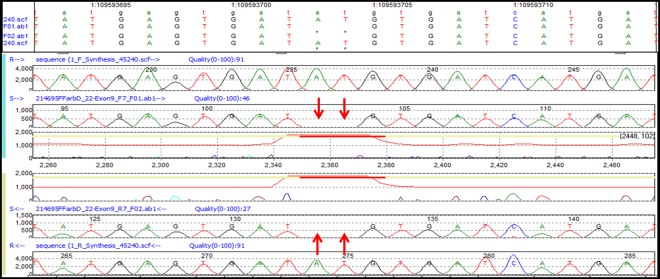
Validation of HaloPlex homozygous deletion (c.*1379_*1380delAT) of Patient 22 in the non-coding exon 9/3’ UTR of *GNAI3* by Sanger Sequencing. In addition to the track annotation panel clearly indicating the deletion at positions 109593703–109593704, the figure shows the forward sequences of the reference (R) and test sample (S) and the reverse sequences. The red arrows point to the deleted nucleotides.

In Fig 5, the red arrows point to the position and nucleotides deleted in Patient 22, and the mutant trace is then aligned to the reference, but in reality the patient’s nucleotide sequence is shifted by two nucleotides from that deletion onward.

### Computational model analyses of the *GNAI3* non-synonymous mutations

**Fig 6:**
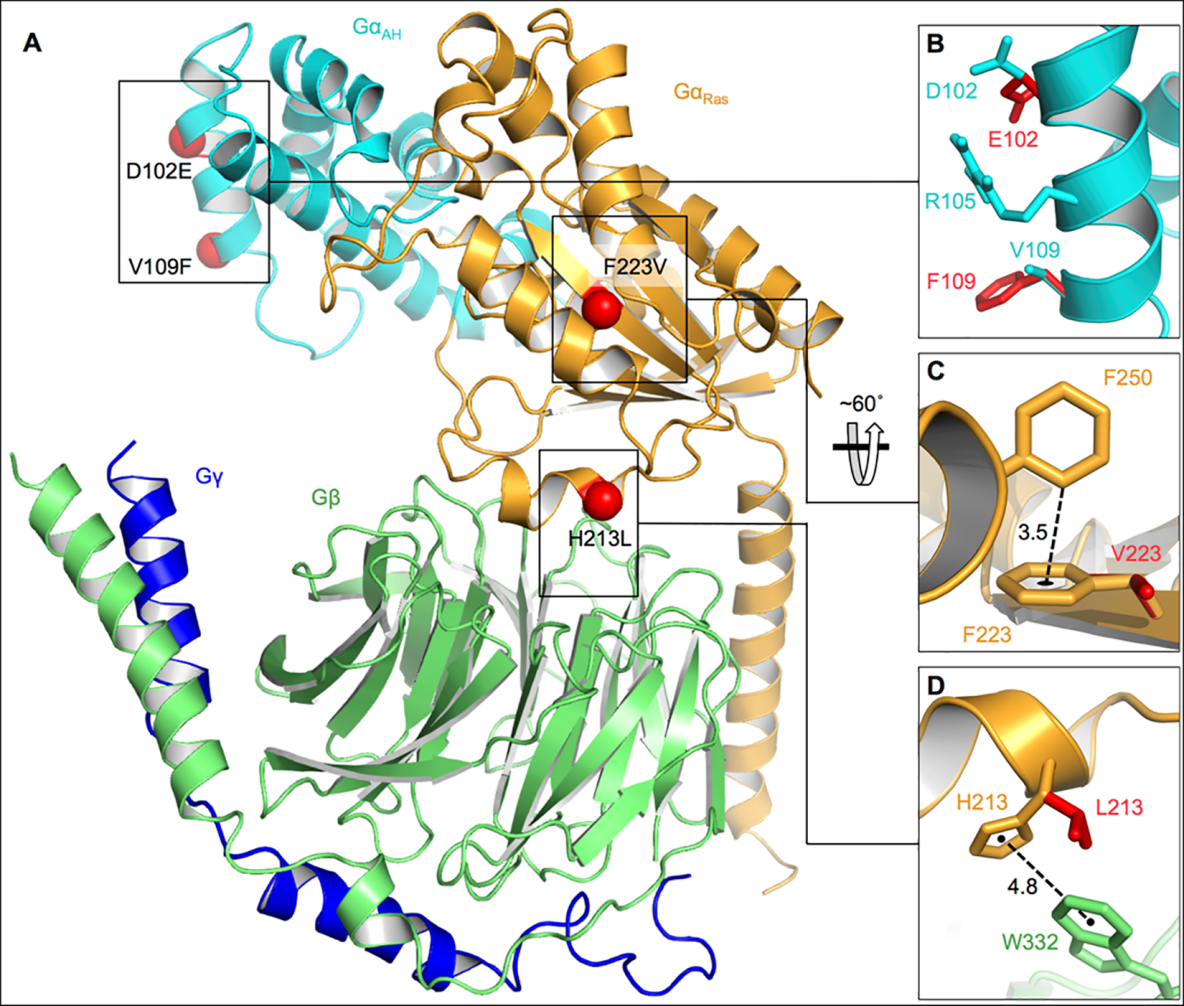
Structural representation of heterotrimeric GNAI3. **A)** In the Gαβγ model, two domains of Gα are depicted: the Gα-helical insertion domain (Gα_AH_) is in cyan and the Ras-like GTPase domain (Gα_Ras_) is in gold; the Gβ subunit is in green, and the Gγ subunit in blue. Sites of observed amino-acid mutations are indicated with red spheres. B) Magnified representation of mutations D102E and V109F as well as of amino-acid R105. Wild-type side chain carbons are in cyan while mutated residues are in red. C) Magnified representation of the “T-shaped” π-stacking interaction between F223 and F250 in wild-type GNAI3 (gold) that is lacking in the F223V mutant (red). The distance from the *ortho* carbon on F250 to the centroid of F223 is 3.5 Å, shown with a dotted black line. D) Magnified representation depicting the “parallel displaced” π-stacking interaction between α-H213 (gold) and β-W332 (green) that is absent in the H213L mutant (red). The distance between the centroids of the aromatic rings is 4.8 Å, shown with a dotted black line.

The crystal structure of heterotrimeric GNAI3 shows the two domains of the Gα subunit: the Gα-helical insertion domain (Gα_AH_) and the Ras-like GTPase domain (Gα_Ras_) (Fig 6A). Amino-acid residues D102 and V109 are on the α-helical insertion domain (Gα_AH_), close to an arginine at position 105 and distal to the interfacial region between Gα_Ras_ and the OA1 GPCR (Fig 6A and 6B). F233 is within the GNAI3 GTPase domain and forms a “T-shaped” π-stacking interaction with-F250 (Fig 6C). H213 is on the Gα and Gβ subunits interface and it engages in a “parallel-displaced” π-stacking interaction with β-W332 (Fig 6D).

Computational model analyses of the D102E, V109G and V109F, F223V and H213L variants were carried out to investigate the structural and functional consequences of these amino-acid changes in GNAI3 (Fig 6A–6D). According to this model, D102E (in Patient 3) and V109G (in Patient 16) or V109F (in Patient 21) have no effect on the binding of GNAI3 to the OA1 GPCR but they may stabilize the α-helix through a distance-enhanced interaction with R105. Variant F223V in Patient 21 increases conformational flexibility not allowing proper pre-organization of the α-subunit of GNAI3, hindering in this way the efficiency of activation for binding either to the GPCR or to GTP [22]. On the other hand, H213L in Patient 7 destabilizes the GNAI3 heterotrimer by abolishing the favorable π-stacking interaction between the Gα and Gβ subunits.

## Discussion

Numerous reports indicating that some persons with ocular albinism have no specific pathogenic mutations in *OA1* [12–14] raised the possibility that a different gene in the same transduction pathway could be responsible for the abnormal visual phenotype of these patients. Since results from our previous studies in mice had shown that: 1) Oa1 activates specifically Gαi3 in its signaling pathway [6]; 2) *Gαi3 -/-* animals have similar RPE abnormalities to those of Oa1-/- mice [7]; and 3) a constitutively active Gαi3 protein corrects the RPE phenotype of *Oa1-/-* mice [23], we investigated whether mutations in the human *GNAI3* gene may cause the ocular albinism phenotype. Using a combination of HaloPlex and MiSeq sequencing, we identified many variants in the *GNAI3* gene that were found only in the DNA of patients diagnosed clinically with ocular albinism but whose DNA had tested negative for OA1 mutations. None of these variants was present in the DNA of control individuals.

For our HaloPlex experiments, we used the *GNAI3* gene sequence from the Ensembl Homo sapiens GRCh37/hg19 as a reference to design all the amplicons. In this Ensembl, *GNAI3* non-coding exon 9/3’UTR was 2,009 bp long. With the 2013 update to Ensemble Homo sapiens GRCh38/hg38, all coding exons and introns of the *GNAI3* gene remained unchanged, but the non-coding exon 9/3’UTR was extended to 25,977 bp. This version of the *GNAI3* gene was used as reference in our genetic screening studies. We identified mutations not only in coding exons 1, 4, and 6, but also in introns, in the 5’ UTR and in the region that we analyzed of the non-coding exon 9/3’UTR. We specifically investigated the two latter regions because several recent studies have shown that mutations in the UTRs are associated with pathogenic changes leading to disease [24–26].

In general, the 5’-UTR contains numerous binding sites for proteins that either repress or promote transcription in response to molecular signals. Mutations in those specific sites lead to alterations in the transcription of the corresponding mRNAs and subsequently in the expression of the encoded proteins, thus causing disease. Eight of the 26 patients studied had one of the six SNVs that we identified in the 5’UTR of *GNAI3* (marked with an asterisk in Table 3); Patients 10 and 26 had two SNVs each. SNVs with assigned identification numbers rs3737182 and rs1279195 were found previously to be involved in the alteration of a transcription factor binding site (NIH SNP Function Prediction program, https://snpinfo.niehs.nih.gov/snpinfo/snpfunc.htm) and in chromatin interactions [OKCAM V2.0 platform http://rhesusbase.org/OKCAM/ [27]]. Only functional studies will determine whether any of the six 5’ UTR mutations that we detected here either decreases the *GNAI3* mRNA and protein levels or produces a non-functional GNAI3 protein that cannot inhibit the traffic of vesicles carrying melanosomal proteins from the TGN to the melanosomes, as we had hypothesized previously [7]. These results would explain the presence of macromelanosomes in the RPE of persons with ocular albinism who don’t have a specific pathogenic mutation in *OA1*.

We identified several non-synonymous variants in exons 1, 4, and 6 of *GNAI3* in the DNA of some of the patients studied (Table 4). Patient 2 presented a homozygous frameshift deletion in exon 1 (c.83delA, E28f), which could have a significant effect on his GNAI3 protein. Since this deletion is in the first exon of the gene, it will alter the first stop codon encountered in the DNA sequence and produce an abnormally short or long not functional polypeptide. In other words, it will most probably cause disease. We carried out computational analyses to understand the structural and functional consequences of the other non-synonymous variants in GNAI3.

It is known that the Gα subunits of G-proteins have two domains: a Ras-like GTPase domain (Gα_Ras_) responsible for anchoring Gα into the GPCR and associating with the β subunit as well as an α-helical insertion domain (Gα_AH_, Fig 6A) [28]. Together, these two domains surround the guanine nucleotide-binding pocket. Formation of a GDP-bound Gαβγ heterotrimer is a prerequisite for activation by the receptor and is followed by GTP exchange and subsequent dissociation of the Gα and Gβγ subunits to effect further signaling. According to the structural model of GNAI3 represented in Fig 6, amino-acid residues D102 and V109 are on the α-helical insertion domain of GNAI3, distal to the interfacial region between Gα_Ras_ and OA1 (Fig 6A and 6B). Thus, the observed mutations D102E (Patient 3), V109G (Patient 16) and V109F (Patient 21) will have no direct impact on the binding of GNAI3 to the GPCR. It is conceivable that independently, mutations D102E and V109F each may stabilize the α-helix through more facile hydrogen bonding and N-H•••π contacts with R105, respectively, which can potentially reduce the binding affinity with OA1 or other proteins through propagation of structural perturbations. However, the energy reduction from these proposed interactions would be counteracted by an entropic penalty associated with freezing out conformational degrees of freedom of flexible side chains. In addition, V109G cannot be rationalized in this way since there would not be a stabilizing interaction between the resulting glycine side chain and R105. The fact that V109F and V109G both serve to induce a disease phenotype provides evidence that the isopropyl side chain of valine is precisely the appropriate size to fill a specific binding pocket between the Gα_AH_ domain and another protein. Considering this possibility, we decided to examine known binding modes of GNAI3 with other compounds. We found that D102 and V109 are removed from the interaction site of Gαi3 proteins with the regulator of G protein signaling (RGS) family, as shown in crystal structures with RGS2, [18] RGS8, [29] and RGS10 [29] (PDB codes: 2V4Z, 2ODE, 2IHB). We propose that these residues are important, not in the activation phase of GNAI3 by OA1, but rather in the regulation of trafficking of melanosomal proteins from the ER/trans-Golgi to the melanosomes [7]. Interestingly, substitution of an aspartic acid for a glutamic acid in other genes also leads to disease. For example, D645E in lysosomal α-glucosidase causes 67% decrease in the activity of the enzyme [30]. The wild-type enzyme is present in various cellular compartments of the glycoprotein-transport pathway (ER, Golgi, and trans-Golgi network), while the mutant is retained in the ER. Since the N-acetylglucosaminyl phosphotransferase responsible for the phosphorylation of the lysosomal α-glucosidase precursor is present in the *cis*-most Golgi cisternae [31], the D645E mutant precursor cannot be phosphorylated. Thus, this mutation accounts in full for defects in transport, phosphorylation, and proteolytic processing of the newly synthesized α-glucosidase precursor and thus causes glycogen-storage disease type II (GSDII) [32]. Similarly, a mutation D180E in the mature lipoprotein lipase (LPL) results in a virtual absence of LPL enzyme activity and LPL enzyme mass in patients affected with a type of familial chylomicronemia [33]. Also, a non-synonymous substitution of valine for glycine, such as the one in Patient 16, has been linked to the clinical features of early-onset familial Alzheimer disease caused by a missense mutation (V717G) in the amyloid β precursor protein [34]. Several diseases have been associated with the substitution of valine for phenylalanine, like V109F present in Patient 21. For example, a recent novel mutation in the Janus activated kinase 2 gene (JAK2 V617F) seems to be prevalent in patients with mesenteric vein thrombosis and myeloproliferative disorders [35].

Our computational model of GNAI3 shows that within its GTPase domain F223 forms a “T-shaped” π-stacking interaction at a distance of 3.5 Å from its centroid to the nearest carbon of F250 (Fig 6C). Density functional theory calculations in the gas phase predict that these types of non-covalent interactions result in roughly 3 kcal mol^-1^ of stabilization relative to infinitely separated monomers [36]. We speculate that this interaction reduces motion and anchors relevant parts of the Gα structure during the conformational changes leading to activation [22]. The F223V mutation, present in Patient 21, does not allow this interaction and thus, the activation of GNAI3, possibly leading to the abnormal phenotypes characteristic of ocular albinism. Interestingly, the same missense mutation in the LIM2 gene, causing F105V in the Lens Intrinsic Membrane Protein 2, is associated with autosomal recessive presenile cataracts [37].

Our computational model also shows that α-H213 is on the interface of the Gα and Gβ subunits and engages in favorable π-stacking with β-W332 that is lost upon mutation to leucine, i.e., H213L in Patient 7 (Fig 6D). “Parallel displaced” π-stacking interactions between benzene and heteroaromatic groups are predicted with density functional theory calculations to produce ~3.5–5.0 kcal mol^-1^ in the gas phase [38] and are considered to play an important role in stabilizing the native structures of proteins [39–41]. A histidine-tryptophan interaction is integral in the folding of microsomal apocytochrome b_5_ and is sufficiently strong to resist disruption in 8 M urea [42]. Coupled cluster calculations have predicted the π-stacking energy to be about 4 kcal/mol, larger than that of histidine-phenylalanine and histidine-tyrosine, presumably because of the larger π system of tryptophan [43]. Thus, we speculate that the interaction of α-H213 with β-W332 is a key factor contributing to the ability of GNAI3 to form a complex with the β-subunit and is therefore necessary for activation of GNAI3 by OA1. There is evidence that π-stacking interactions in RNA binding proteins are evolutionarily conserved [44], which further stresses their importance in maintaining global protein structure and function [45, 46]. Substitutions of histidine for leucine in other genes have also been found to cause disease. In the Gγ-globin gene, this substitution is associated with methemoglobinemia and cyanosis in the newborn [47].

Human non-coding exons, disabled by frameshifts and premature stop codons, are embedded within 5' and 3’ UTRs and they can have different parts of their sequence differentially spliced in alternative transcripts with regulatory function. Together with the UTRs, non-coding exons are usually longer than coding exons. *GNAI3* non-coding exon 9 is a part of the 3’UTR and it spans 25,977 bp.

In general, 3′UTRs are implicated in regulation of gene expression. They contain both binding sites for miRNAs as well as silencer regions for regulatory proteins. By binding to these specific sites, miRNAs decrease mRNAs’ levels through translational repression or mRNA cleavage [48], and repressor proteins inhibit mRNA expression [49]. SNVs, deletions or insertions have been shown to impact regulation by altering the miRNA binding sites [50]. Moreover, many 3’ UTRs also contain AU-rich elements (AREs). Dysregulation of ARE-binding proteins due to mutations can lead to diseases such as cancer, hematopoietic malignancies and leukemogenesis [51, 52]. Thus, SNVs, deletions, or insertions may affect the 3’ UTR regulatory sequences of *GNAI3* and play a role in the expression of the ocular albinism phenotype. Indeed, we have identified 41 variants in the first 2,109 bp of *GNAI3* non-coding exon 9/3’ UTR, 27 of which are SNVs and 14 indels.

Several of the identified SNVs [e.g., c.*1734C>T (rs2301229), c.*1902T>A (rs3525), c.*1940T>C (rs41280328)] are predicted to have an effect on chromatin interactions and miRNA binding sites (OKCAM V2.0 platform and NIH SNP Function Prediction program). We also used the RegRNA 2.0 web server to identify functional RNA motifs in the *GNAI3* 3’UTR and confirmed that many detected variants affect directly these regulatory elements. For example, four SNVs: 1) c.*1696T>G (Patients 1, 11, and 21), at Chr1-109594018, 2) c.*1697T>G (Patient 7) and 3) c.*1697 T>A (Patient 24), both at Chr1-109594019, as well as 4) c.*1698T>G (Patients 7 and 12), at Chr1-109594020, respectively (Table 5), disrupt the regulatory element GAIT (Gamma interferon activated inhibitor of translation), which is important in the silencing of translation of several genes [53]. GAIT is a specific binding site of hsa-miR-548, a miRNA known to be involved in the regulation of actin cytoskeleton, MAPK signaling pathway, ubiquitin mediated proteolysis and of several types of cancer [54]. Moreover, the deletion c.*1934delG at position 109594256 of Chr1 in Patient 3 (Table 6) disrupts not only the regulatory element GAIT but also the miR139-5p binding site, which is important for mRNA translational silencing of genes [55].

Further, both a *GNAI3* 3' UTR regulatory element, SECIS, and the binding site for miR-744-5p located within the SECIS sequence, are disrupted in Patients 11 and 13 by the deletion c.*1654delA at Chr1 position 109593976 (Table 6). This deletion may deregulate expression of miR-744-5p and may be associated with the abnormal phenotype of ocular albinism. Interestingly, miR-744-5p is expressed specifically in AMD patients and is a promising biomarker for the rapid diagnosis of AMD [56].

Other variants affecting *GNAI3* 3’ UTR sequences are found in the two different deletions c.*1378_*1379delGA (Chr.1:109593700) and c.*1379_*1380delAT (Chr.1:109593703) identified in the binding site of hsa-miR-144 in Patients 11, 13, 22, and 24 (Table 6). This binding site has been predicted to be present in the 3’ UTR of the *NRF2* gene and has been demonstrated to be essential for the regulation of the NRF2 pathway in human RPE cells. The NRF2 pathway plays a central role in the response of the RPE to oxidative stress, a key factor associated with AMD [57]. In addition, hsa-miR-144 has been associated with regulation of the insulin receptor substrate 1 (IRS1) that controls the metabolic state of the cell [58] and of the *MITF* gene, which encodes the microphthalmia-associated transcription factor [59]. *MITF* controls the RPE expression of OA1 [60], which in turn is involved in the development of melanocytes and, together with GNAI3, in the regulation of melanogenesis. It is possible that the two deletions in the binding site of hsa-miR-144 affect the hsa-miR-450b-5p binding site, which is only 2 nucleotides downstream. miR-450b-5p acts as a strong repressor of Pax6, a major regulator of eye development [61].

Besides all the variants in exonic and untranslated regions of *GNAI3* described above, we found 462 additional SNVs and indels distributed along the introns of *GNAI3*. Introns, that constitute about 26% of the human genome [62], are integral to gene expression and regulation. Splice site mutations occur during the processing of precursor mRNA into mature mRNA. These mutations may lead to retention of large segments of intronic DNA by the mRNA or to entire exons being spliced out of the mRNA, and could result in production of non-functional proteins. For example, an intronic splicing mutation found in the *OA1* gene of a patient with ocular albinism created a new acceptor splice site in intron 7 of *OA1* and, in addition, it activated a cryptic donor-splice site causing the inclusion of a large intronic fragment between exons 7 and 8. The aberrant splicing generated a novel splicing enhancer motif, ASF/SF2 that favored the transcription of the mutated mRNA, explaining the abnormal phenotype [63]. Moreover, intronic deletions resulting in elimination of potential recognition sites for splicing factors located within the deleted sequence, even when leaving the canonical splice site intact, may result in skipping of exons [64]. Other intronic variants may interfere with some essential roles played by introns in a wide range of gene expression regulatory functions such as nonsense mediated decay [65] and mRNA export [66]. Given the large number of intronic variants that we found in the *GNAI3* gene, their analyses will constitute the subject of a future study that will include functional verification of the possible effects of some of them.

In conclusion, this is the first NGS-based genetic study of persons with ocular albinism who do not have a mutation in the *OA1* gene. HaloPlex target enrichment led to the identification and validation of many novel variants/mutations in *GNAI3* coding and non-coding exons, introns, and the 3’ and 5’ UTRs. Some of these mutations are pathogenic and can result in a non-functional GNAI3 protein leading to the characteristic phenotype of ocular albinism. Thus, *GNAI3* is a second gene, in addition to *OA1*, responsible for this disease. Genetic screening of *GNAI3* and eventually of other genes corresponding to proteins (as yet not identified) involved in the *OA1* signaling cascade may benefit individuals who present the ocular albinism phenotype but do not have any mutations in OA1. This implication has obvious benefits in the development of future therapies.
